# Measurement uncertainty of ISO 17511:2020 compliant and globally standardized PT/INR test results

**DOI:** 10.1016/j.rpth.2026.103385

**Published:** 2026-02-02

**Authors:** Micehelle P. van der Helm, Claudia J.J. van Rijn, Shanti S. Baktawar, Charmane F. Abdoel, Steve Kitchen, Michelle Bryant, Paula Brown, Armando Tripodi, Erica Scalambrino, Marigrazia Clerici, Petra Herbel, Anja Jünschke, Piet Meijer, Anne Stavelin, Craig Thelwell, Christa M. Cobbaert, Antonius M.H.P. van den Besselaar

**Affiliations:** 1 Department of Clinical Chemistry and Laboratory Medicine, Coagulation Reference Laboratory (CRL), Leiden University Medical Center (LUMC), Leiden, The Netherlands; 2 Sheffield Haemophilia and Thrombosis Centre, Royal Hallamshire Hospital, Sheffield, United Kingdom; 3 IRCCS Ca’ Granda Maggiore Hospital Foundation, Angelo Bianchi Bonomi Hemophilia and Thrombosis Centre, Milano, Italy; 4 Roche Diagnostics GmbH, Mannheim, Germany; 5 External quality Control of diagnostic Assays and Tests (ECAT) Foundation, Voorschoten, The Netherlands; 6 The Norwegian Organisation for Quality Improvement of Laboratory Examinations, Bergen, Norway; 7 Therapeutic Reference Materials, Medicines and Healthcare products Regulatory Agency, National Institute for Biological Standards and Control, Potters Bar, United Kingdom

A reference measurement system (RMS) for the prothrombin time (PT)/International Normalized Ratio (INR) test has been proposed by the IFCC-SSC/ISTH working group on PT/INR standardization based on a single international reference preparation (IRP) for thromboplastin [1]. Calibration hierarchies according to ISO 17511:2020 have been developed for point-of-care whole blood and wet laboratory citrated plasma samples [2]. The RMS, which is IFCC-SSC/ISTH endorsed [1,2], consists of the harmonized manual tilt tube technique (MTT) and the WHO 6th International Standard (IS) for Thromboplastin, Human, Recombinant (coded 24/114), designated in ISO 17511:2020 as a “component of reference measurement system (paragraph [3.41])” and hereinafter referred to as IRP 24/114. IRP 24/114 is expected to replace the current 5th IS for thromboplastin, rabbit, plain (coded RBT/16) and the 5th IS for thromboplastin, human, recombinant (coded rTF/16) from 2026 onwards. In the RMS [1,2] 4 calibration labs form the calibration network. They perform the harmonized MTT for INR value assignment with the primary IRP characterized by an International Sensitivity Index (ISI) and Mean Normal PT (MNPT) [3]. The coagulation reference laboratory at Leiden University Medical Center is one of the calibration labs.

Analytical performance specifications have been defined for the RMS based on Milan model 1: criteria built on outcome-based performance specification [4]. A relative total expanded error of ± 20% was derived from an INR therapeutic target value of 2.5 and a measurement error not exceeding ±0.5 INR. It was stated that a total standard uncertainty (expressed as coefficient of variation) of the INR should not exceed 10% [2]. The uncertainty budget of the RMS (performed by the calibration labs) was set as a rule of thumb at 1/3 of the 10%, being 3.3% [2,5]. That leaves theoretically 1/3 of the uncertainty budget for the IVD-manufacturers and 1/3 for the end-user. The aim of this study is to assess the standard MU of the RMS. The data of the multicenter study performed in 2024, which are published separately, have been used.

Due to the complexity of the reaction equation of the PT measurement with multiple individual coagulation factors with unknown concentrations and exact relations, there is no chemical reaction and rate equation to be determined with individual coagulation factor concentrations. Therefore, it is not realistic to establish a mechanistic kinetic model for MU determination taking into account the coagulation cascade. ISO/TS 20914:2019 provides an equation for INR MU determination [6]. However, the equation does not take into account uncertainty in the ISI and has been criticized for not correctly applying the GUM [7] principles [8,9]. Other methodologies have also been used for INR uncertainty determinations, including top-down and bottom-up approaches and Monte Carlo simulations [[9], [10], [11], [12]]. Here, we have calculated the standard MU of the INR as proposed earlier by Van den Besselaar et al. [13]. This MU calculation approach is an empirical model in log-space making use of the standard deviation (SD)_logINR_ with propagation of error formulas [14] (Equation 1) according to GUM [7]/ EURACHEM/CITAC guideline principles [15]:

(1) $$SDlogINR=\sqrt{\left\{\frac{{\left(ISI*SDlogPT\right)}^{2}}{n}+\frac{{\left(ISI*SDlogMNPT\right)}^{2}}{m}+{\left(logR*{SD}_{ISI}\right)}^{2}\right\}}$$

where R = PT/MNPT is the PT ratio, ISI is the International Sensitivity Index for the specific thromboplastin/measurement system, SD_logINR_, SD_logPT_, SD_logMNPT,_ and SD_ISI_ are the standard deviations for log INR, log PT, log MNPT, and ISI, and n and m are the number of PT and MNPT determinations, respectively. The equation comprises MU components of the ISI, MNPT, and PT, representing the ISI reproducibility (determined between lab), the MNPT intermediate precision (same operator over time), and the PT repeatability (same operator multiple measurements). The ISI is an external quantity, and the uncertainty of the assigned value is determined by the results from multiple calibration labs. The assigned average ISI is used by the operator, who will measure the PT of an unknown plasma and then convert the result to INR. The MNPT, determined as the geometric mean of the PTs of citrated plasmas from each of 20 healthy individuals, is derived from prior calibration studies by the same operator. Even though, for INR measurements in reality the components of Equation (1) (ISI, MNPT and PT) are not entirely independent, we have decided to not include extra terms in Equation (1) for covariance. Since the ISI is determined with fresh plasmas by multiple laboratories, and the MNPT is determined from multiple calibration studies, we consider the measurements with the lyophilized plasmas as independent measurements.

Both for the current human recombinant-based IRP (rTF/16) and IRP 24/114 the MU-values in terms of SD_logINR_ have been calculated (Table). The CI is calculated using *k = 2* as the coverage factor to obtain an approximate confidence level of 95%. The standard MU is expressed in a %ru_ref_ (relative uncertainty) value based on SD_logINR_ (Table), considering that for small SDlog_INR_ and under a log-normal distribution the approximation of %ru_ref_ **≈** 100xSD_logINR_ holds. Lyophilized plasmas (A, B, C, and D) are used for MU determination. Lyophilized materials allow for replicate measurements on multiple days with identical material. Plasma A has an INR of ∼1.0 (normal plasma, outside the therapeutic range), plasma B ∼2.0-2.3, plasma C ∼3.0-3.5, and plasma D ∼4.2-5.6 (outside the therapeutic range). For IRP 24/114 only 2 MNPT (m = 2) by the same operator in our laboratory have been determined up till now, hence limiting this approach. In the future when more MNPT determinations have been performed with IRP 24/114, the MU can be even more accurately established. However, since the SD_logMNPT_ is a small contributor to the MU it is not expected that the overall MU will change drastically with more MNPT determinations. The largest contributor to the MU is the SD_ISI_, since this value is calculated based on the results from the 4 calibration labs of the multicenter study in 2024 (the reproducibility component). It should be noted that SD_ISI_ is only based on four calibration laboratories (with 2 operators in each laboratory). Thus, SD_ISI_ and SD_logMNPT_ are estimated from small N, which introduces uncertainty into SDlogINR. Nevertheless, comparing the SD_ISI_ of rTF/16 (obtained in the previous multicenter study in 2018 with 20 laboratories using a non-harmonized MTT [16]) with the SD_ISI_ of IRP 24/114 it is evident that the SD has reduced by twofold, resulting also in lower SD_logINR_ values and a median %ru_ref_ change from 5.8% (median rTF/16 %ru_ref_-value of INR obtained from Table data) to 2.7% (median IRP 24/114 %ru_ref_-value of INR obtained from Table data). These improvements are attributed to the harmonization efforts of the MTT [1], as well as the fact that the SD_ISI_ of IRP 24/114 was determined with only one previous IRP (rTF/16), in contrast to the SD_ISI_ of rTF/16 (and IRPs before that), which was (were) determined with two previous IRPs (rabbit and human recombinant) historically (see reference [2] and Figure in the reference). Only for lyophilized plasma A rTF/16 demonstrates a lower SD_logINR_ than IRP 24/114. Note also that the lyophilized plasmas used in this work are non-commutable, considering the difference in 95% CI lower and upper bounds of rTF/16 and IRP 24/114 (Table).

**Table:** Standard MU (%ru_ref_) of INR determined with rTF/16 and IRP 24/114 in terms of SD_logINR_ (approximation: %ru_ref_ ≈ 100xSD_logINR_)

IRP	Plasma	Mean PT (s)	SD_logPT_	Mean MNPT (s)	SD_logMNPT_	ISI	SD_ISI_	Mean INR	SD_logINR_	95% CI of INR	%ru_ref_ of INR
rTF/16	Lyo A	12.17 (*n* = 10)	0.021	12.17 (m = 6)	0.014	1.11	0.063	1.00	0.010	0.98-1.02	1.0
rTF/16	Lyo B	25.25 (*n* = 10)	0.0090	12.17 (m = 6)	0.014	1.11	0.063	2.25	0.047	2.05-2.47	4.7
rTF/16	Lyo C	36.64 (*n* = 10)	0.011	12.17 (m = 6)	0.014	1.11	0.063	3.40	0.070	2.95-3.91	7.0
rTF/16	Lyo D	56.46 (*n* = 10)	0.020	12.17 (m = 6)	0.014	1.11	0.063	5.49	0.098	4.52-6.67	9.8
IRP 24/114	Lyo A	10.69 (*n* = 10)	0.016	11.28 (m = 2)	0.016	1.08	0.030	0.94	0.014	0.92-0.97	1.4
IRP 24/114	Lyo B	19.80 (*n* = 10)	0.016	11.28 (m = 2)	0.016	1.08	0.030	1.84	0.022	1.76-1.92	2.2
IRP 24/114	Lyo C	29.38 (*n* = 10)	0.016	11.28 (m = 2)	0.016	1.08	0.030	2.81	0.032	2.64-3.00	3.2
IRP 24/114	Lyo D	41.70 (*n* = 10)	0.017	11.28 (m = 2)	0.016	1.08	0.030	4.10	0.042	3.78-4.46	4.2

The 95% CI is calculated using mean_logINR_ ± 2 × SD_logINR_. N.B. the natural logarithm (ln) is used in the calculations. IRP, international reference preparation; INR, international normalized ratio; ISI, international sensitivity index; MNPT, mean normal prothrombin time; PT, prothrombin time; ru, relative uncertainty; SD, standard deviation.

The range of %ru_ref_ values for IRP 24/114 were 1.4 to 4.2 % (Table), showing increasing %ru values with increasing INRs. This finding is in line with the MU estimations with the Monte Carlo simulations [10] and previous work [17]. For INRs of lyophilized plasmas B and C (within the therapeutic window), we obtain 2.2 % and 3.2 %, respectively (Table). This is within the predefined uncertainty budget of <3.3% for the RMS at the top of the traceability chain and also in line with the between-operator CV values of 3.2% in the multicenter study for lyophilized plasmas B and C. The data show that this bottom-up approach with SD_logINR_ corresponds well to the top-down approach with between-operator CVs. From the MU datapoints also a precision profile (according to CLSI EP17) is constructed, fitted with a power function, and shown in relation to the uncertainty budget and calibration hierarchy of plasma INR measurements in Figure. The power function can be used to calculate the MU at any INR-value between 1.0-4.0. It should be mentioned; however, that the MU here only focuses on the primary reference thromboplastin (IRP 24/114) at the start of this new RMS [2]. After some years, when a new batch of a secondary reference thromboplastin will be established, an updated MU-value must be determined. When primary and secondary reference thromboplastins are both in place, the MU for the calibration labs will likely not fit the theoretically predefined 3.3% MU budget. Subsequently, the MU budget will have to be divided differently between calibration labs, IVD-manufacturers, and end-user laboratories to still remain at 10% INR total standard MU at the end-user level. Since the estimated standard MU within the therapeutic window presented here is around 2-3% for IRP 24/114 (the primary reference thromboplastin), the total calibration-level contribution (with primary and secondary reference thromboplastin) may be closer to half of the total MU budget than to one-third, especially at therapeutic INRs. It is therefore pragmatically proposed to give the primary and secondary reference thromboplastins together ½ of the MU budget of 10%, which leaves then also ½ to the IVD-industry, and end-users together (illustrated in Figure).

**Figure:**
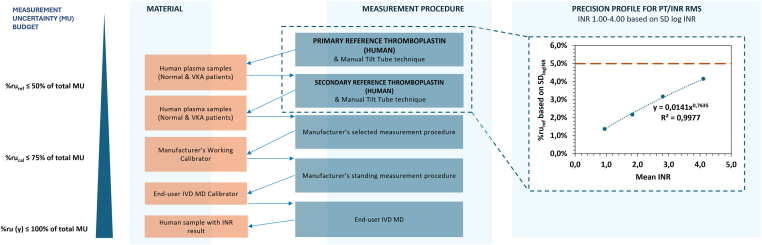
Precision profile of the RMS for the standard MU (%ru_ref_ in terms of SD_logINR_) against the mean INR for IRP 24/114, shown in relation to the calibration hierarchy (for detailed INR calibration hierarchies see reference [2]) and measurement uncertainty budget of plasma INR measurement. The data are fitted with a power function. The updated MU budget for primary and secondary reference thromboplastin with harmonized MTT as half of 10% for the calibration laboratory reports is depicted with a dashed orange line. INR, international normalized ratio; IRP, international reference preparation; MU, measurement uncertainty’ RMS, reference measurement system.

Reflecting on these findings, it remains essential to thoroughly evaluate the RMS implementation to assess the full impact of the INR uncertainty defined by the RMS and how it propagates through the metrological traceability chain. Following successful integration of the RMS with the single human-recombinant IRP, a comprehensive validation of the resulting uncertainty levels at the end-user level is required. Also important to note is the fact that the MU estimates in this work are derived at the calibration laboratory level using lyophilized, non-commutable plasmas. They characterize the contribution of the IRPs to the overall uncertainty budget but do not directly quantify the MU of INR results in routine laboratories using patient samples.

A RMS has been proposed for the PT/INR test comprising the harmonized MTT with IRP 24/114. Calculation of the MU using the SD_logINR_ formula based on propagation of errors and using components for reproducibility (ISI), repeatability (PT), and intermediate precision (MNPT) gives values <3.3% for INR-values in the therapeutic range (INR 2-3) for lyophilized plasmas. This is in line with between-operator CVs (3.2%) found in the multicenter study (2024). Based on these findings the MU meets the analytical performance specifications criteria (<3.3% for the calibration labs) based on outcome (Milan model 1) for the INR [2]. These results are a first step towards global PT/INR standardization by laying the foundation at the calibration laboratory level for an interlaboratory variation (CV%) at the end-user level of <10%. The results have to be combined with manufacturer calibrations and inter-laboratory studies. Evaluation of end-user uncertainty within 10% is required after successful implementation of the new RMS.
